# Takotsubo cardiomyopathy following aeromonas hydrophila septic shock after traumatic injury: a case report

**DOI:** 10.3389/fcvm.2026.1781127

**Published:** 2026-03-12

**Authors:** Xu Zeng, Yi Chen, Wang Du, Kechun Zhou

**Affiliations:** Department of Emergency Medicine, The Fifth Affiliated Hospital of Wenzhou Medical University, Lishui Central Hospital, Lishui Hospital of Zhejiang University, Lishui, Zhejiang, China

**Keywords:** aeromonas hydrophila, echocardiography, magnetic resonance imaging (MRI), septic cardiomyopathy, takotsubo cardiomyopathy (TTS)

## Abstract

**Background:**

Aeromonas hydrophila is a Gram-negative bacillus commonly found in aquatic environments. It is capable of causing skin and soft tissue infections, and in immunocompromised individuals, it may lead to severe systemic infections and septic shock. Takotsubo cardiomyopathy (TTS) is a rare cardiac syndrome typically precipitated by acute stressors.

**Case introduction:**

This report describes a case of a patient who experienced cardiac arrest following trauma sustained in a road traffic accident, complicated by lower limb infection. The infection progressed rapidly, culminating in cardiac arrest. Subsequent investigations supported a diagnosis of TTS occurring in a multifactorial physical stress milieu, including traumatic injury and rapidly progressive soft-tissue infection due to Aeromonas hydrophila with septic shock. The patient was successfully treated with targeted antibiotic therapy, surgical intervention, and individualized haemodynamic support with cautious, congestion-aware fluid management, and was ultimately discharged in a stable condition.

**Conclusion:**

Aeromonas hydrophila infection with septic shock may be one component of a multifactorial physical stress response; together with traumatic injury and severe pain from tissue injury and necrosis, it may precipitate Takotsubo syndrome through acute sympathetic activation and a catecholamine surge. Echocardiography and cardiac magnetic resonance imaging can support the diagnosis. In this case, levosimendan was used as an adjunctive inotropic strategy; however, no causal inference or therapeutic efficacy can be concluded from a single case report.

## Introduction

Aeromonas hydrophila is a Gram-negative bacillus commonly found in freshwater and brackish environments. It is associated with a variety of clinical manifestations, including gastroenteritis, wound infections, and sepsis. This pathogen can infect not only immunocompromised individuals but also those with normal immune function (1). Most cases of necrotising soft tissue infections caused by A. hydrophila have been associated with traumatic injuries exposed to aquatic environments (2). However, to date, there have been no documented cases of stress-induced cardiomyopathy secondary to soft tissue infection, and reports of endocarditis caused by A. hydrophila remain exceedingly rare (3).

Takotsubo cardiomyopathy (TTS) is a transient cardiac dysfunction characterised by apical ballooning and hypokinesis of the anterior septal and apical regions in the absence of obstructive coronary artery disease. These abnormalities typically resolve spontaneously, with restoration of normal left ventricular function (4, 5). TTS is often triggered by emotional or physical stress, including infections, surgical procedures, and trauma (6). Here, we report a case of TTS after traumatic injury complicated by rapidly progressive lower-limb soft-tissue infection due to Aeromonas hydrophila and subsequent septic shock. After inadequate wound debridement at a local hospital, the soft-tissue infection progressed rapidly; in temporal association, the patient developed TTS and cardiac arrest. The patient recovered following prompt resuscitation, early surgical intervention, and targeted antimicrobial therapy.

## Case report

A 70-year-old male with a medical history of hypertension, diabetes mellitus, and rectal carcinoma was admitted following altered mental status for 4 h and generalised pain for 2 days. Two days prior to admission, he had fallen into a roadside ditch during a motor vehicle accident and sustained multiple injuries, predominantly involving the left lower limb and right shoulder. He was transported by emergency medical services to a local hospital where he was diagnosed with degloving injury and multiple fractures of the lower limb, and subsequently underwent emergency debridement and vacuum sealing drainage (VSD) surgery.

On postoperative day two, the patient developed acute chest tightness. Despite symptomatic treatment (details not available), he deteriorated and experienced cardiopulmonary arrest. He was intubated and received mechanical ventilation with 30 min of cardiopulmonary resuscitation (CPR), after which spontaneous circulation and respiration were restored. The patient was transferred to our tertiary care centre for further management.

**Figure 1:**
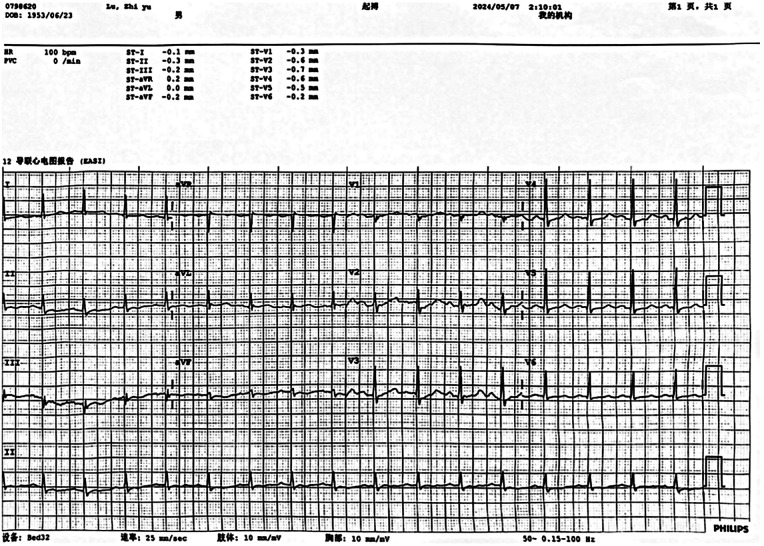
Results of the patient's ECG.

**Figure 2:**
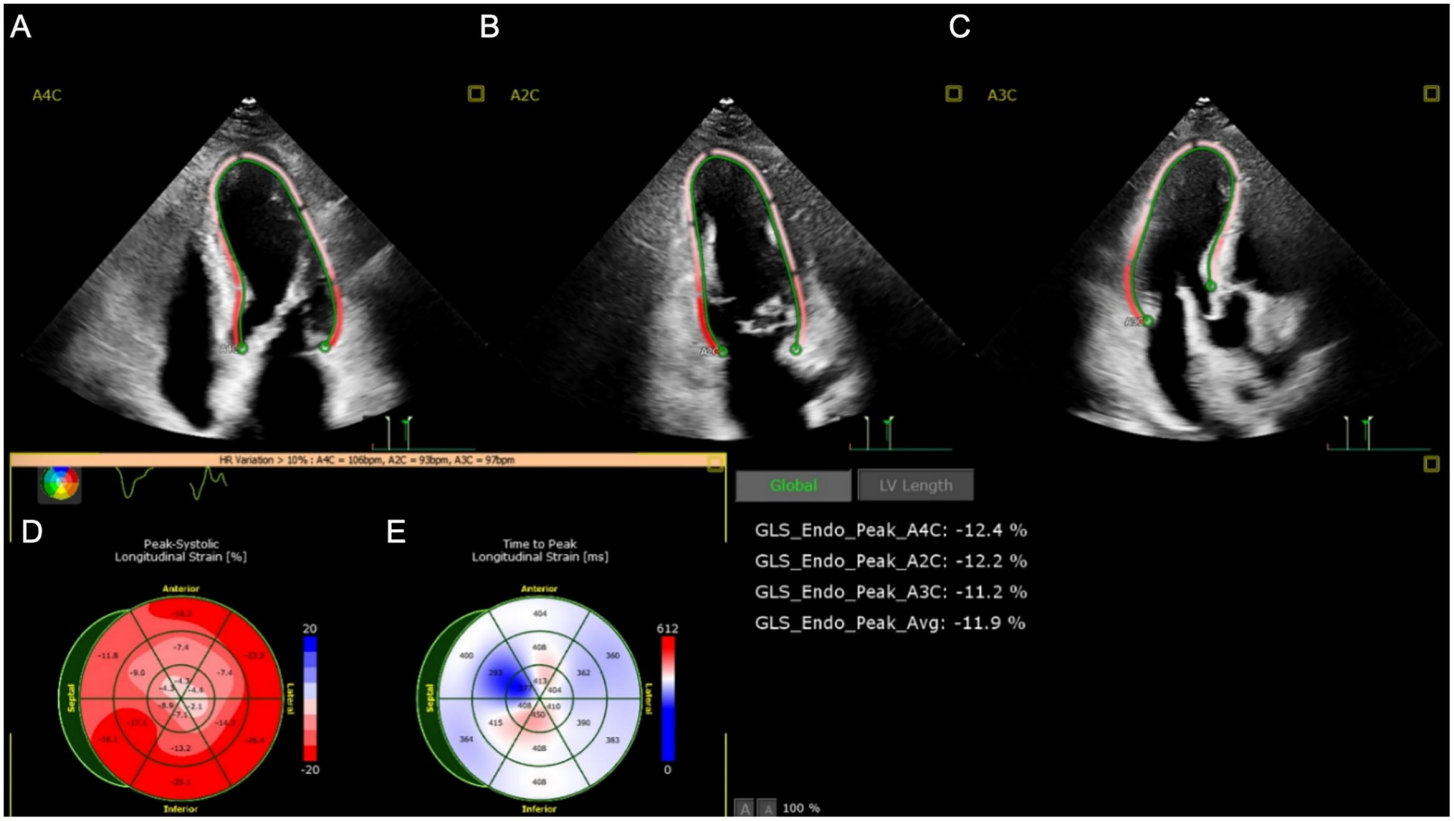
The results of the patient's echocardiogram. **(A,B,C)** Ultrasound images of the patient's heart in the 4-chamber, 2-chamber, and 3-chamber views. **(D,E)** Graph of the Peak-Systolic Longitudinal Strain of the heart.

**Figure 3:**
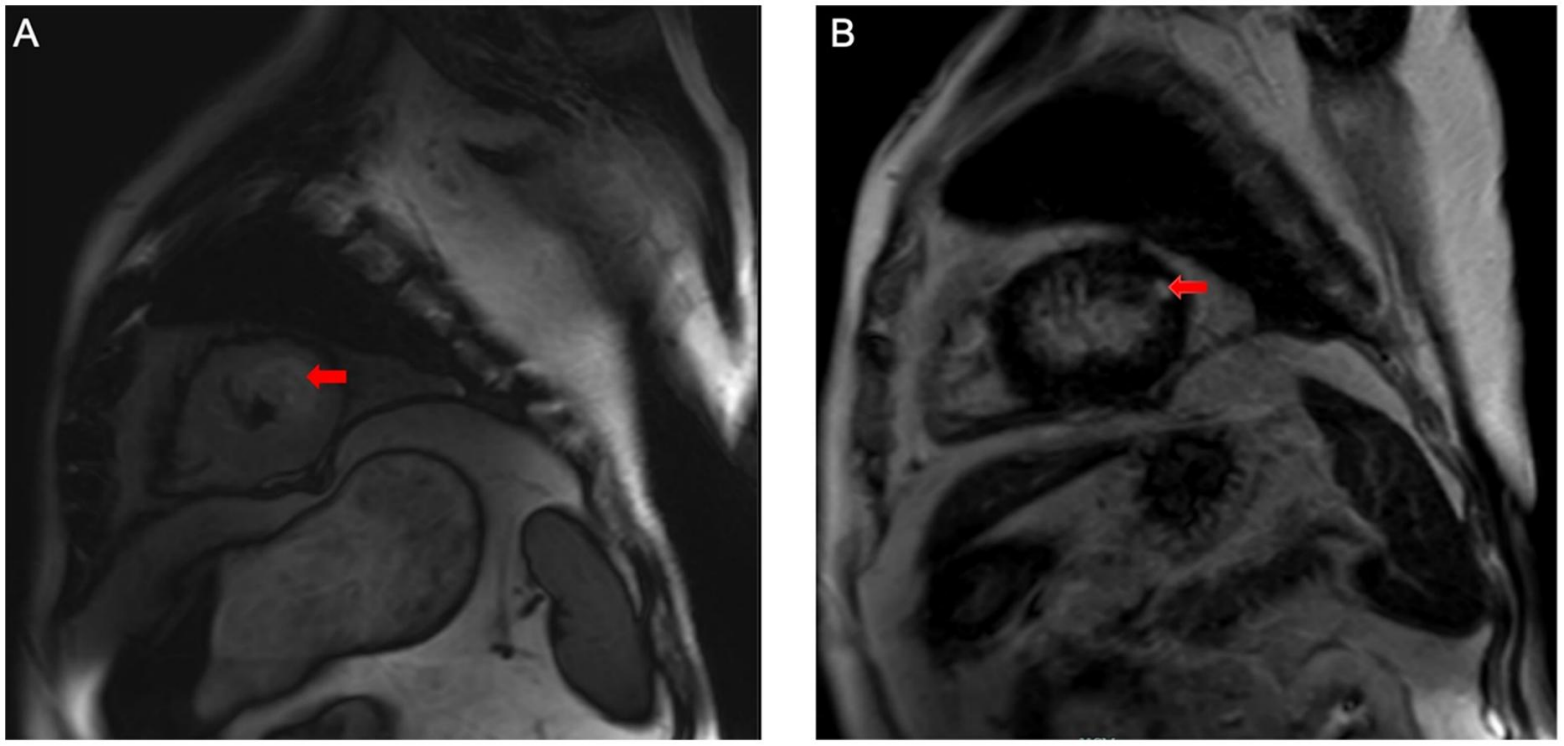
The results of the patient's cardiac MRI. **(A)** On the T2-STIR sequence, there is mild edema in the apical region of the heart (red arrow). **(B)** On the late gadolinium enhancement sequence, mild fibrosis can be observed in the apical region of the heart (red arrow).

**Figure 4:**
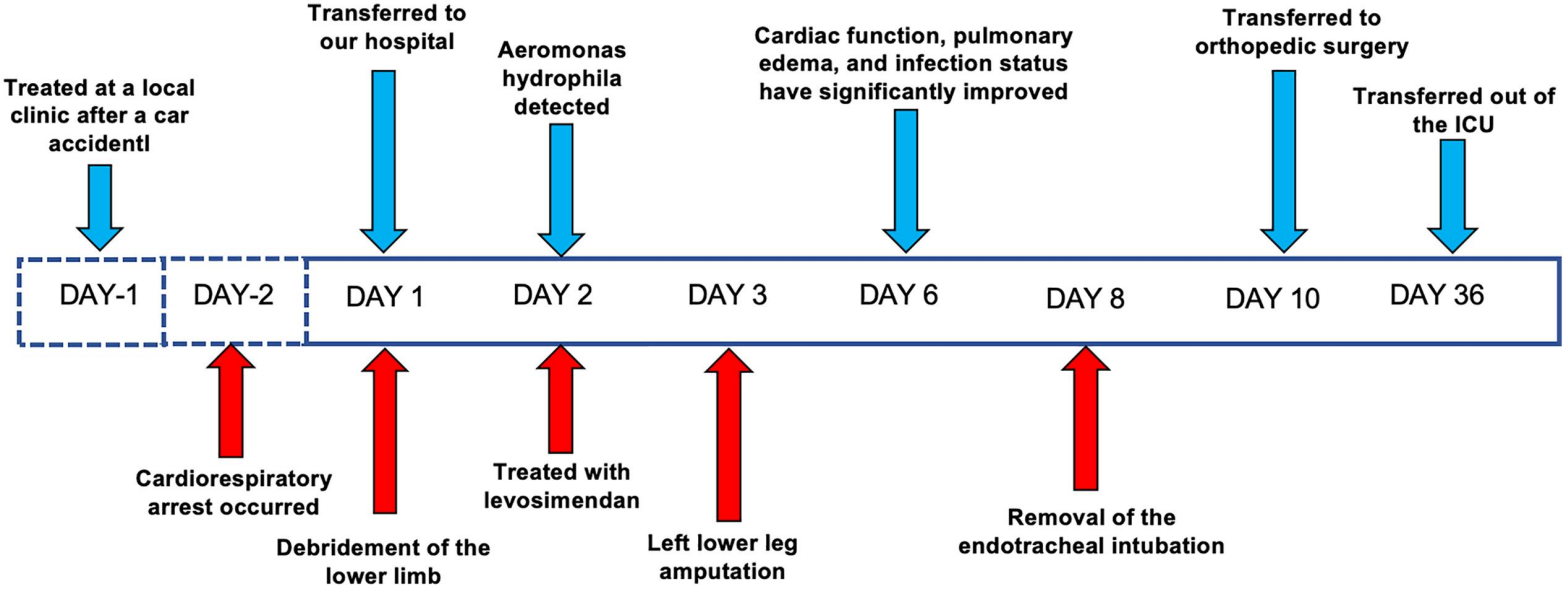
Patient's treatment course.

Upon admission, laboratory results revealed marked abnormalities: C-reactive protein (CRP) was 178.48 mg/L; white blood cell count 19.0 × 10^9^ /L; neutrophil ratio 85.5%; red blood cell count 2.24 × 10^12^ /L; haemoglobin 71 g/L. Liver and renal function tests showed elevated ALT (467 U/L), AST (658 U/L), creatinine (95 μmol/L), and blood glucose (10.02 mmol/L). Cardiac markers indicated elevated brain natriuretic peptide (BNP) at 724.2 pg/mL and procalcitonin at 8.04 ng/mL. Coagulation parameters were deranged: prothrombin time was 16.7 s; D-dimer 16.26 mg/L; antithrombin III 61.5%. Myocardial injury markers were significantly raised: creatine kinase 1,162 U/L, myoglobin 754.4 ng/mL, troponin I 20.400 ng/mL. Arterial blood gas analysis (FiO_2_100%) showed PaCO_2_of 44.4 mmHg, PaO_2_of 82.3 mmHg, and lactate of 10.3 mmol/L.Thoracic computed tomography angiography (CTA) revealed atherosclerosis with mild stenosis in some coronary arteries, apical wall thickening and dilatation, bilateral pulmonary infiltrates indicative of infection and pulmonary oedema, and multiple rib fractures. Electrocardiogram showed sinus rhythm with ST-segment depression in leads V2–V4 (Figure 1). Transthoracic echocardiography demonstrated left atrial enlargement, reduced left ventricular systolic function, apical chamber dilatation, and global hypokinesis, with an end-diastolic volume (EDV) of 106.51 mL, end-systolic volume (ESV) of 77.52 mL, and ejection fraction (EF) of 27% (Figure 2). The initial diagnosis was stress-induced cardiomyopathy, cardiogenic shock, septic shock, acute respiratory distress syndrome (ARDS), left lower limb degloving injury, multiple lower limb fractures, and pelvic fracture. At admission, the patient had a body temperature of 36.7 °C, heart rate of 90 bpm, respiratory rate of 31 breaths/min, and blood pressure of 138/89 mmHg (maintained with high-dose vasoactive agents). His body weight was 80 kg, his APACHE II score was 15 and the SOFA score was 8. After admission, the patient immediately underwent debridement of the left lower extremity and vacuum sealing drainage (VSD). Cefoperazone–sulbactam (3.0 g every 12 h) was administered for anti-infective therapy. Levosimendan (0.15 μg/kg/min) was infused continuously for 24 h to augment cardiac function. Norepinephrine was initiated at 0.8 μg/kg/min and titrated to maintain a MAP ≥ 65 mmHg, with fluid therapy guided by PiCCO using dynamic and volumetric parameters, while closely monitoring extravascular lung water and pulmonary vascular permeability to avoid fluid overload. Crystalloids were used as first-line resuscitation fluid, and the crystalloid-to-colloid (albumin) proportion and cumulative fluid balance were strictly controlled given the high risk of worsening pulmonary oedema. Blood and sputum cultures were obtained to identify the source of infection. Given severe hypoxemic respiratory failure with bilateral pulmonary infiltrates/pulmonary oedema in the setting of marked LV systolic dysfunction (EF 27%) and shock, venoarterial ECMO (VA-ECMO) was considered as rescue support for combined circulatory failure and refractory hypoxemia. However, the patient's family declined ECMO because of concerns regarding risks and costs. Prone-position ventilation was therefore initiated as an adjunctive oxygenation strategy (rather than a replacement for mechanical circulatory support), and the ventilator settings were adjusted (AC/PC mode; respiratory rate 12 /min; pressure control 15 cmH2O; PEEP 8 cmH2O; FiO2 80%; inspiratory time 0.9 s). On day two, blood and wound cultures identified Aeromonas hydrophila. Based on susceptibility testing, the antibiotic regimen was changed to piperacillin–tazobactam (4.5 g every 8 h). By day three, the wound showed progressive necrosis and poor infection control. As cardiopulmonary function had stabilised, the patient underwent left lower limb amputation and wound reconstruction. Following comprehensive treatment, the patient showed significant clinical improvement by day six. Pulmonary oedema resolved, infection was controlled, and oxygenation improved. The endotracheal tube was successfully removed on day eight. On day ten, the patient was transferred to the orthopaedic ward for further surgical management. He was readmitted to the ICU due to gastrointestinal bleeding postoperatively. Cardiac magnetic resonance imaging (MRI) was performed during the ICU stay (Figure 3). The patient was discharged from the ICU on day 36 and began a rehabilitation programme, including prosthesis implantation and physiotherapy. He was ultimately discharged in stable condition. Figure 4 illustrates the clinical timeline of disease progression and major interventions.

## Discussion

In clinical practice, Takotsubo syndrome (TTS) occurring in the context of Aeromonas hydrophila infection is exceedingly rare. The uniqueness of the present case lies in the development of a systemic inflammatory response and subsequent TTS following A. hydrophila infection after traumatic exposure to contaminated water. Although both the traffic accident and the infection could have served as stressors, literature indicates that trauma-induced TTS typically manifests within several hours of the triggering event (7, 8). In this case, the patient did not exhibit acute symptoms such as chest tightness on the day of the accident. However, as the infection progressed and developed into septic shock the following day, the patient began to experience a constellation of symptoms culminating in cardiopulmonary arrest—findings consistent with the clinical presentation of TTS.

TTS is typically precipitated by emotional or physical stress. Acute critical illness, trauma, and neurological disorders (e.g., traumatic brain injury) are recognised triggers. In this case, several physical stressors coexisted—including traumatic injury, severe pain/tissue injury from rapidly progressive soft-tissue necrosis, cardiopulmonary resuscitation, and subsequent A. hydrophila–related septic shock—which likely acted synergistically to precipitate TTS.Accordingly, A. hydrophila–related septic shock should be interpreted as a contributing trigger within a multifactorial stress response rather than the sole precipitant. Under Sepsis-3, sepsis is defined as life-threatening organ dysfunction caused by a dysregulated host response to infection; sepsis and particularly septic shock represent major physical stressors that may precipitate TTS.

Although the mechanisms of Takotsubo syndrome (TTS) remain incompletely defined, current consensus supports a brain–heart axis in which acute physical stressors—such as traumatic injury, severe pain/tissue necrosis, cardiopulmonary resuscitation, and infection with septic shock—activate central autonomic networks, leading to hyperactivation of the sympathetic nervous system and a surge in catecholamines. Excess catecholamines, acting through beta adrenergic receptor signaling, can provoke myocardial injury and reversible stunning, partly through activation of cAMP protein kinase A signaling that promotes intracellular calcium overload, metabolic and mitochondrial dysfunction, and oxidative stress. In parallel, adrenergic and inflammatory pathways may promote coronary microvascular dysfunction and endothelial injury, further amplifying regional wall-motion abnormalities (9). Therefore, in this patient, septic shock is best interpreted as a contributory trigger and physiological amplifier within a multifactorial catecholaminergic stress response, rather than the dominant or sole precipitant of TTS. Catecholamine excess is a key mechanism in TTS. In this patient, a marked endogenous catecholamine surge was plausible given the concurrence of major physical stressors (trauma, severe pain/tissue necrosis, septic shock, and cardiopulmonary resuscitation). The potential contribution of exogenous catecholamines should also be considered, as vasopressor support required high-dose norepinephrine for shock management (up to 0.8 μg/kg/min), which may have amplified catecholaminergic exposure and contributed to the development and/or severity of TTS. However, because the initial cardiac event occurred at the referring hospital and detailed pre-transfer catecholamine timing/doses were unavailable, causality attributable to exogenous norepinephrine cannot be inferred and is acknowledged as a limitation.

In the setting of septic shock, TTS may be precipitated or exacerbated through several converging mechanisms, including sympathetic overactivation with catecholamine excess, inflammation-mediated endothelial and microvascular dysfunction, and haemodynamic instability (6). Sepsis-induced sympathetic overactivation leads to surges in catecholamines such as norepinephrine and adrenaline. These high catecholamine levels have direct cardiotoxic effects, increase myocardial energy demand, promote calcium overload, and impair contractility—resulting in the hallmark apical ballooning pattern seen in TTS. Furthermore, the cytokine storm often associated with sepsis—involving a massive release of both pro-inflammatory (e.g., IL-2, IL-4, IL-8, IFN-γ, TNF-α) and anti-inflammatory (e.g., IL-10) mediators—can dysregulate vascular tone, impair microcirculation, and aggravate myocardial injury, all of which contribute significantly to TTS pathogenesis. Simultaneously, sepsis-induced hypotension and haemodynamic instability may increase coronary microvascular resistance and impair myocardial perfusion, further contributing to TTS onset.

As a Gram-negative bacillus, A. hydrophila is capable of inducing sepsis through endotoxin-mediated activation of the systemic inflammatory response. Its lipopolysaccharide (LPS) can stimulate widespread cytokine release (e.g., TNF-α, IL-1, IL-6) and trigger catecholamine secretion via adrenal medullary activation. This infectious stress response may directly impair myocardial contractility and lead to the characteristic “apical ballooning” pattern. Although reports of A. hydrophila-induced TTS are sparse, other pathogens with similar pathogenic mechanisms offer indirect evidence. Clinical cases have documented Clostridium difficile and Klebsiella infections as triggers of TTS (10). In those instances, patients presented with transient apical hypokinesis and normal coronary angiography findings, consistent with TTS. The proposed mechanism also involved endotoxin-driven inflammatory and catecholamine-mediated myocardial injury.

The high mortality associated with acute-phase TTS highlights the importance of early recognition and intervention. Studies report an in-hospital mortality rate of up to 4.2% for TTS (11), underscoring the need for rapid diagnosis to improve prognosis. Transthoracic echocardiography remains the most accessible and pivotal diagnostic tool; however, coronary angiography is essential to exclude obstructive coronary artery disease (12). In this case, echocardiography revealed typical apical hypokinesis and reduced left ventricular ejection fraction, with coronary angiography confirming the absence of significant coronary obstruction, supporting a diagnosis of TTS (13).

LV systolic dysfunction in the setting of sepsis may occur in both sepsis-induced cardiomyopathy (SICM) and TTS. Either entity can result in low cardiac output, pulmonary edema and/or shock, and their phenotypes may overlap; thus, a comprehensive diagnosis should integrate echocardiographic wall-motion patterns, right ventricular (RV) involvement, cardiac biomarker profiles, and CMR findings. To clarify this differential diagnosis in the context of septic shock, the key distinguishing features of TTS vs. SICM and their application to this patient are summarized in Table 1. TTS is characterized by stress-related, reversible myocardial stunning, with imaging features that emphasize regional, circumferential wall-motion abnormalities extending beyond a single coronary artery territory; the typical patterns include apical or mid-ventricular dysfunction with relative basal hyperkinesis (13). By contrast, SICM represents a reversible myocardial depressive state: echocardiography more commonly demonstrates diffuse/global systolic impairment, frequently accompanied by RV dysfunction and/or diastolic dysfunction. Troponin elevation and electrocardiographic changes are generally nonspecific, and increased troponin more often reflects sepsis-related myocardial injury or supply–demand mismatch rather than a specific etiology; cardiac function usually improves with infection control and hemodynamic optimization (14). With respect to biomarkers, TTS often shows a rise in troponin that is not fully proportional to the degree of LV systolic dysfunction, whereas BNP/NT-proBNP levels tend to be relatively higher. However, this “disproportion” may be attenuated in septic shock, post–cardiopulmonary resuscitation states, or shock, so biomarkers should be considered supportive clues rather than decisive evidence. In addition, RV involvement may occur in TTS but is not obligatory. From a differential diagnostic perspective, diffuse biventricular involvement favors SICM, whereas circumferential involvement predominantly affecting specific LV segments supports a TTS phenotype (15). CMR is particularly critical for differentiating the two. In acute TTS, myocardial edema corresponding to the wall-motion abnormality is commonly observed (e.g., on T2-STIR imaging or elevated T1/T2 mapping). Classic descriptions emphasize the absence of late gadolinium enhancement (LGE) consistent with ischemic infarction; however, recent CMR studies suggest that when imaging is performed early after symptom onset or when more sensitive interpretation thresholds are applied, a subset of TTS cases may exhibit mild/diffuse or reversible LGE. Therefore, “complete absence of LGE” should not be regarded as an absolute requirement. Conversely, prominent, segmental LGE conforming to a coronary distribution should raise concern for alternative diagnoses such as acute myocardial infarction or myocarditis (16). For SICM, CMR data remain relatively limited; prior reports more often describe diffuse edema without a specific LGE pattern and without the segmental distribution typical of TTS (17).

**Table 1:** Comparative features of TTS vs. SICM.

Domain	Typical TTS	Typical SICM	Findings in this patient
Echocardiography	Transient left ventricular dysfunction is typically characterized by focal apical wall-motion abnormalities. The regional wall-motion abnormalities usually extend beyond the territory supplied by a single coronary artery and may be accompanied by right ventricular involvement.	Predominantly global ventricular systolic dysfunction (often with LV dilatation); typically no regional WMA pattern like apical ballooning (though overlap/“Takotsubo-like” patterns can occur)	In this patient, left ventricular systolic function was reduced with apical cavity dilatation and predominantly apical focal wall-motion abnormalities.
LVEF	Acutely reduced (variable severity), expected to recover as part of “transient” dysfunction	Depressed EF with LV dilatation; reversible in survivors	EF 27% (EDV 106.51 mL; ESV 77.52 mL)
Reversibility	Typically improves over days–weeks; recovery of LV function supports diagnosis retrospectively	Classically reversible in 7–10 days in survivors	“LV function improved markedly over a short period” after infection control and circulatory support
Biomarkers	Troponin/CK moderately elevated in most; BNP/NT-proBNP elevation common	Troponin elevation is common in septic shock; BNP may rise but is not specific for SICM	Troponin I 20.400 ng/mL, BNP 724.2 pg/mL
Cardiac MRI	Edema in dysfunctional segments is common; “classic” descriptions emphasize absence of infarct-pattern LGE, but mild/low-intensity and potentially reversible LGE can be seen in a subset	Available CMR in septic shock/sepsis-associated cardiomyopathy supports an inflammatory phenotype (increased T1/T2/ECV in those with impaired LVEF); LGE pattern is not typically diagnostic	T2-STIR: mild apical edema; LGE: mild apical delayed enhancement/fibrosis

In the present case, echocardiography demonstrated predominantly apical focal wall-motion abnormalities rather than diffuse global depression, with no clear evidence of RV involvement. Coronary CTA showed only mild atherosclerosis and no culprit lesion corresponding to the wall-motion abnormality. During hospitalization, LV function improved markedly over a short period following infection control and circulatory support. CMR revealed a small amount of apical edema with mild delayed enhancement (fibrotic change). Given that early LGE may be present in a subset of TTS cases and typically does not follow a classic ischemic distribution, the overall evidentiary chain is more consistent with a stress-triggered TTS phenotype in a critically ill patient with coexisting trauma and septic shock than with typical SICM.

Current guidelines for the management of TTS emphasise early identification and removal of triggering factors (16). In this case, given the left lower limb wound as the infection source and poor response to multiple debridement procedures, early amputation was performed. Once the causative organism was identified, antibiotic therapy was promptly adjusted. Beyond source control and antimicrobial therapy, TTS management is primarily supportive, with beta-blockers recommended over routine inotropes to avoid exacerbating catecholamine toxicity. However, some studies suggest that levosimendan may be beneficial in selected cases to improve myocardial contractility and haemodynamic stability. Levosimendan exerts its effects by binding to cardiac troponin C in a calcium-dependent manner, enhancing myofilament calcium sensitivity. It also opens ATP-sensitive potassium channels, resulting in vasodilation that reduces both preload and afterload. In addition, it dilates the pulmonary and coronary arteries, alleviating right ventricular afterload and pulmonary hypertension, thereby mitigating myocardial ischaemia during septic shock (18). These mechanisms are consistent with previous reports by Schlürmann et al., where levosimendan was successfully used in TTS management (19). Given the marked cardiac dysfunction and pulmonary oedema, volume resuscitation was individualized and haemodynamic-guided using PiCCO, integrating dynamic indices of fluid responsiveness with volumetric and perfusion parameters, and serial EVLWI and PVPI monitoring; crystalloids were used as first-line fluids, the crystalloid to albumin proportion and cumulative fluid balance were strictly controlled to avoid worsening pulmonary oedema, and plasma was reserved for correction of clinically significant coagulopathy or bleeding rather than routine volume expansion.As Takotsubo-specific evidence does not mandate a single fluid strategy, volume management should be tailored to perfusion and congestion, consistent with sepsis guidance favouring dynamic assessment over static parameters and with the use of EVLWI/PVPI to warn against excessive volume expansion in patients at risk of lung oedema. Although venoarterial extracorporeal membrane oxygenation (VA-ECMO) was initially considered to manage low cardiac output and severe pulmonary oedema, the patient's family declined this intervention due to financial concerns. In the context of limited treatment options, levosimendan was cautiously administered as adjunctive therapy. Although the patient ultimately achieved favourable outcomes, we acknowledge that levosimendan is not part of the standard treatment for TTS, and its efficacy requires further validation through prospective clinical studies.

## Conclusion

Aeromonas hydrophila infection complicated by septic shock may represent one component of a multifactorial physical stress response that can precipitate Takotsubo syndrome, alongside traumatic injury and severe pain or tissue necrosis. Distinctive imaging features on echocardiography and cardiac magnetic resonance (CMR) differentiate TTS from septic cardiomyopathy, and recognising these specific patterns is essential for the early and accurate diagnosis of TTS. Supportive care with prompt source control, targeted antimicrobial therapy, and individualized haemodynamic support including cautious, congestion-aware fluid therapy remains the cornerstone of management; in this case, levosimendan was selected as adjunctive non-catecholamine inotropic support to minimize adrenergic stimulation, but the evidence for levosimendan in Takotsubo syndrome is limited mainly to small observational studies and case reports, and randomized trials are still needed.

## Data Availability

The original contributions presented in the study are included in the article/Supplementary Material, further inquiries can be directed to the corresponding authors.
